# Alcohol Intake and Endogenous Hormones in Pre- and Postmenopausal Women: Findings from the UK Biobank

**DOI:** 10.1158/1055-9965.EPI-21-0789

**Published:** 2021-10-04

**Authors:** Sandar Tin Tin, Timothy J. Key, Gillian K. Reeves

**Affiliations:** Cancer Epidemiology Unit, Nuffield Department of Population Health, University of Oxford, Oxford, UK.

## Abstract

**Background:**

Alcohol intake may influence breast cancer risk in women through hormonal changes, but the evidence to date is inconclusive. We investigated cross-sectional associations between habitual alcohol intake and serum concentrations of testosterone, sex hormone binding globulin (SHBG), insulin-like growth factor-1 (IGF-1), and estradiol (premenopausal women only) in UK Biobank.

**Methods:**

We included 30,557 premenopausal and 134,029 postmenopausal women aged between 40 and 69 years when recruited between 2006 and 2010. At their initial assessment visit, habitual alcohol intake was assessed using a touchscreen questionnaire, and serum hormone concentrations were assayed. Multivariable linear regression analysis was performed.

**Results:**

Per 10 g/day increment in alcohol intake, testosterone concentration was 3.9% [95% confidence intervals (CI): 3.3%–4.5%] higher in premenopausal women and 2.3% (1.8%–2.7%) higher in postmenopausal women (*P*_heterogeneity_ < 0.0001); SHBG concentration was 0.7% (0.2%–1.1%) higher in premenopausal women and 2.4% (2.2%–2.6%) lower in postmenopausal women (*P*_heterogeneity_ < 0.0001); and IGF-1 concentration was 1.9% (1.7%–2.1%) lower in premenopausal women and 0.8% (0.6%–0.9%) lower in postmenopausal women (*P*_heterogeneity_ < 0.0001). In premenopausal women, there was no significant overall association of alcohol with estradiol but a positive association was observed in the early and mid-luteal phases: 1.9% (95% CI: 0.2%–3.6%) and 2.4% (95% CI: 0.7%–4.2%) higher, respectively.

**Conclusions:**

This study confirms significant but modest associations between alcohol intake and hormones, with evidence of heterogeneity by menopausal status.

**Impact:**

The findings facilitate better understanding of whether alcohol intake influences hormone concentrations, but further work is necessary to fully understand the mechanisms linking alcohol with cancer risk.

## Introduction

Alcohol use is prevalent in many countries globally (1) and is a leading risk factor for several diseases including cancer (2). Alcoholic beverages have been judged to be “carcinogenic to humans” (3), but the underlying mechanisms are not fully understood. Potential mechanisms include actions of acetaldehyde, inhibition of DNA methylation, oxidative stress, and changes in metabolism, immune function, and hormones (4, 5).

Sex hormones and growth factors play a role in carcinogenesis, probably by promoting cell proliferation and therefore increasing the chances of random genetic errors occurring and being replicated (6). Circulating estrogens, androgens, and insulin-like growth factor 1 (IGF-1) have been associated with an increased risk of breast cancer in women, whereas sex hormone-binding globulin (SHBG) is inversely associated with the risk (7–10). Breast cancer is also related to alcohol intake (11, 12), suggesting that hormones, if influenced by the latter, may at least in part explain this effect.

Alcohol intake has been associated with sex hormones and growth factors (7, 9, 13). In general, a positive association was observed with estrogens and androgens, and an inverse association with IGF-1, but the results to date have been less conclusive for hormones in premenopausal women due to fewer data being available as well as the fluctuations of hormone concentrations across the menstrual cycle.

We therefore investigated, using data from UK Biobank, the associations between habitual alcohol intake and serum concentrations of testosterone, SHBG and IGF-1 in both pre- and postmenopausal women, and the association of alcohol with estradiol in premenopausal women only. As far as we are aware, this is the largest study on this topic, including over 30,000 premenopausal and 134,000 postmenopausal women.

## Materials and Methods

### Data source

We analyzed data from UK Biobank (project reference number 3248, approved August 2013; ref. 14).

### Study setting

UK Biobank is a prospective cohort study involving about 500,000 adults aged between 40 and 69 years when recruited in 2006 to 2010. At the initial assessment visit, information was collected on socio-demographics, lifestyle and psychosocial factors, and health status, using a self-administered touchscreen questionnaire (15). Physical measurements were undertaken, and blood and urine samples were collected. Dietary intake was also assessed in a subsample of the participants who were recruited in 2009 to 2010, using the Oxford WebQ, a web-based validated 24-hour dietary assessment questionnaire (16).

### Study participants included

This analysis included premenopausal women, who reported they had not had their menopause, i.e., periods stopped, and were younger than 50 years of age, and postmenopausal women, who reported they had gone through menopause, or were 55 years or older, or reported a bilateral oophorectomy. We excluded women who reported being pregnant or currently using hormone therapy [oral contraceptive pills (OCP) or hormone replacement therapy (HRT)], had a prior diagnosis of cancer (except for nonmelanoma skin cancer), or had missing data on time since last menstrual period (premenopausal women), concentrations of one or more hormones or SHBG, or alcohol intake (Supplementary Fig. S1).

### Alcohol intake

Habitual alcohol intake (main exposure of interest) was assessed in all participants using the touchscreen questionnaire. Women were asked how frequently they drank alcohol, and current drinkers were asked about the amount of red wine, white wine or champagne, fortified wine, beer or cider, spirits or liqueurs, and other alcoholic drinks they would drink in an average week or month. Alcohol intake in units per day was calculated by summing the average individual drinks per day, assuming that a glass of red or white wine (six glasses in a standard bottle) contains 1.5 units of pure alcohol, a pint of beer contains 2.5 units, and a glass of fortified wine (12 glasses in an average bottle), a measure of spirits (25 standard measures in a normal-sized bottle) or a glass of other alcoholic drinks contains 1 unit (17). The units were then converted to grams assuming that one unit equals 8 g. Intakes of alcohol from wine (red, white or fortified), beer or cider, and spirits or liqueur were also calculated.

In a subsample of participants, alcohol intake in the 24 hours before blood sampling (24-hour alcohol intake) was assessed at their initial assessment visit using the Oxford WebQ questionnaire. These women were asked to recall if they consumed alcohol in the previous 24 hours, and if so the amount of red wine, rosé wine, white wine, fortified wine, beer or cider, spirits, and other alcoholic drinks consumed. Twenty-four hour alcohol intake in grams was calculated as described above.

### Hormone assays

Hormones and SHBG were measured in blood samples collected at recruitment, using chemiluminescent immunoassays. Detailed information on the assay data has been reported elsewhere (18, 19). The coefficients of variation derived from the internal quality control data are also presented in Supplementary Table S1. Concentrations of free testosterone and free estradiol were calculated based on the law of mass action (20).

Some women had missing assay results for a given hormone due to a very low serum concentration (6% and 18%, respectively, for testosterone in pre- and postmenopausal women; <1% for SHBG and IGF-1; and 20% for estradiol in premenopausal women). These women were included in the analyses by setting the concentration at three quarters of the minimum reportable value. The minimum reportable values were 0.35 nmol/L for testosterone, 0.33 nmol/L for SHBG, 1.30 nmol/L for IGF-1 and 175 pmol/L for estradiol (18). Note that the expected median concentration of estradiol is ∼150 pmol/L in the early follicular phase (21). The estradiol data were not available for postmenopausal women because almost all of them had a concentration below the minimum reportable value of 175 pmol/L. A small proportion of women (<1%) had missing assay results for SHBG due to a very high serum concentration, and for these women the concentration was set at the maximum reportable value of 242 nmol/L (18).

### Statistical analysis

Analyses were undertaken separately for pre- and postmenopausal women (at recruitment). STATA 16 (StataCorp) was used for all analyses.

Hormone concentrations were logarithmically transformed. In premenopausal women, concentrations were then standardized for day of the menstrual cycle with residuals from the mean for each day as described previously (7). Multivariable regression analysis was performed to compare geometric mean concentrations across the categories of habitual alcohol intake. The multivariable models were adjusted for age at recruitment (in 2-year categories for premenopausal women and in 5-year categories for postmenopausal women), ethnicity (White, Black, Asian, and mixed/others), smoking (never, past, current ≤10 cigarettes/day, current >10 cigarettes/day), body mass index (BMI; <22.5 kg/m^2^, 22.5–24.9 kg/m^2^, 25–27.4 kg/m^2^, 27.5–29.9 kg/m^2^, 30–34.9 kg/m^2^, ≥35 kg/m^2^), age at menopause (i.e., when periods stopped; <45 years, 45–49 years, 50–54 years, ≥55 years; postmenopausal women only), time of day of sample collection (hourly from 9:00 am to 8:00 pm) and quintiles of Townsend deprivation index (measure of material deprivation calculated based on postcode and data from the 2001 Census). The impact on the main findings of adjustment for other variables such as intakes of meat, fish, fruit and vegetable, parity, and time since last OCP/HRT use was also assessed in sensitivity analyses.

In women who reported drinking alcohol, trends in hormone concentrations and 95% confidence intervals (CI) per 10 g/day of habitual alcohol intake were estimated using the mean alcohol intake within each category of habitual alcohol intake. The multivariable models were adjusted as described above, and the *P*_trend_ and *P*_heterogeneity_ were calculated.

Similar analyses were undertaken for intakes of alcohol from different drinks (wine, beer or cider, and spirits or liqueur), and these models were also adjusted for the intake of alcohol from other alcoholic drinks. Subgroup analyses were undertaken by smoking status at recruitment (never, past, and current) and by age groups (≤45 vs. >45 years in premenopausal women and ≤60 vs. >60 years in postmenopausal women). In premenopausal women, subgroup analyses were also undertaken by phase of the menstrual cycle. As time since last menstrual period was assessed using the question “How many days since your last menstrual period?” it was assumed that the days were counted from the last day of the most recent period and that the period lasted for three days [the 3-day period was chosen because this provided the distribution of estradiol concentrations across cycle phases similar to that observed previously (7)]. Cycle phases were defined by forward dating as: early follicular = days 0–5, late follicular = days 6–10, mid-cycle = days 11–14, early luteal = days 15–18, mid-luteal = days 19–24, and late luteal = days ≥25 (7). Sensitivity analyses were undertaken, which excluded women with self-reported endocrine disorders, those with irregular menstrual cycles (premenopausal women only) at recruitment and those with a concentration of testosterone or estradiol (premenopausal women only) which was below the reportable range.

In order to assess whether 24-hour alcohol intake was more predictive of serum hormone concentrations, the regression coefficients and 95% CI per 10 g of 24-hour alcohol intake were estimated in a subsample of women who completed the Oxford WebQ questionnaire on the day of recruitment. Subgroup analyses were also undertaken by time of day of blood collection (noon or earlier vs. after noon).

## Results

In total, 30,557 premenopausal and 134,029 postmenopausal women were included, of whom 28,180 and 120,309, respectively, reported drinking alcohol. Table 1 presents characteristics of the study participants.

**Table 1.** Participant characteristics.

	Premenopausal		Postmenopausal	
	All (*N* = 30,557)	Current drinkers (*N* = 28,180)	All (*N* = 134,031)	Current drinkers (*N* = 120,309)
Age at recruitment (years), mean (SD)	44.7 (2.7)	44.7 (2.7)	60.5 (5.3)	60.4 (5.3)
White, %	90.8	93.1	95.5	96.7
Townsend deprivation scores^a^, median (IQR)	−1.8 (4.5)	−1.9 (4.3)	−2.3 (3.9)	−2.4 (3.7)
Habitual alcohol intake (g/day), mean (SD)	10.7 (12.8)	11.6 (12.9)	9.4 (11.4)	10.4 (11.6)
Current smoker, %	11	11.2	7.9	7.9
Body mass index (kg/m^2^), mean (SD)	26.3 (5.2)	26.2 (5.2)	27.3 (5.1)	27.2 (5.0)
Presence of self-reported endocrine disorder(s), %	6.7	6.4	14.1	13.2
Age at menopause (years), mean (SD)	—	—	49.5 (5.7)	49.6 (5.6)
Total testosterone (nmol/L), median (IQR)	1.12 (0.71)	1.12 (0.71)	0.85 (0.74)	0.86 (0.73)
Calculated free testosterone (pmol/L), median (IQR)	12.5 (10.3)	12.5 (10.2)	10.5 (10.8)	10.5 (10.7)
SHBG (nmol/L), median (IQR)	62.9 (38.3)	63.3 (37.8)	53.5 (33.6)	53.6 (33.2)
IGF-1 (nmol/L), median (IQR)	23.4 (6.9)	23.4 (6.8)	20.0 (7.1)	20.1 (7.1)
Total estradiol (pmol/L), median (IQR)	343.9 (368.1)	347.2 (364.8)	—	—
Calculated free estradiol (pmol/L) median (IQR)	4.07 (4.30)	4.04 (4.19)	—	—

Abbreviations: IQR, interquartile range; SD, standard deviation.

^a^Negative values indicate relatively more affluent areas.

### Total and calculated free testosterone

**Figure 1.**
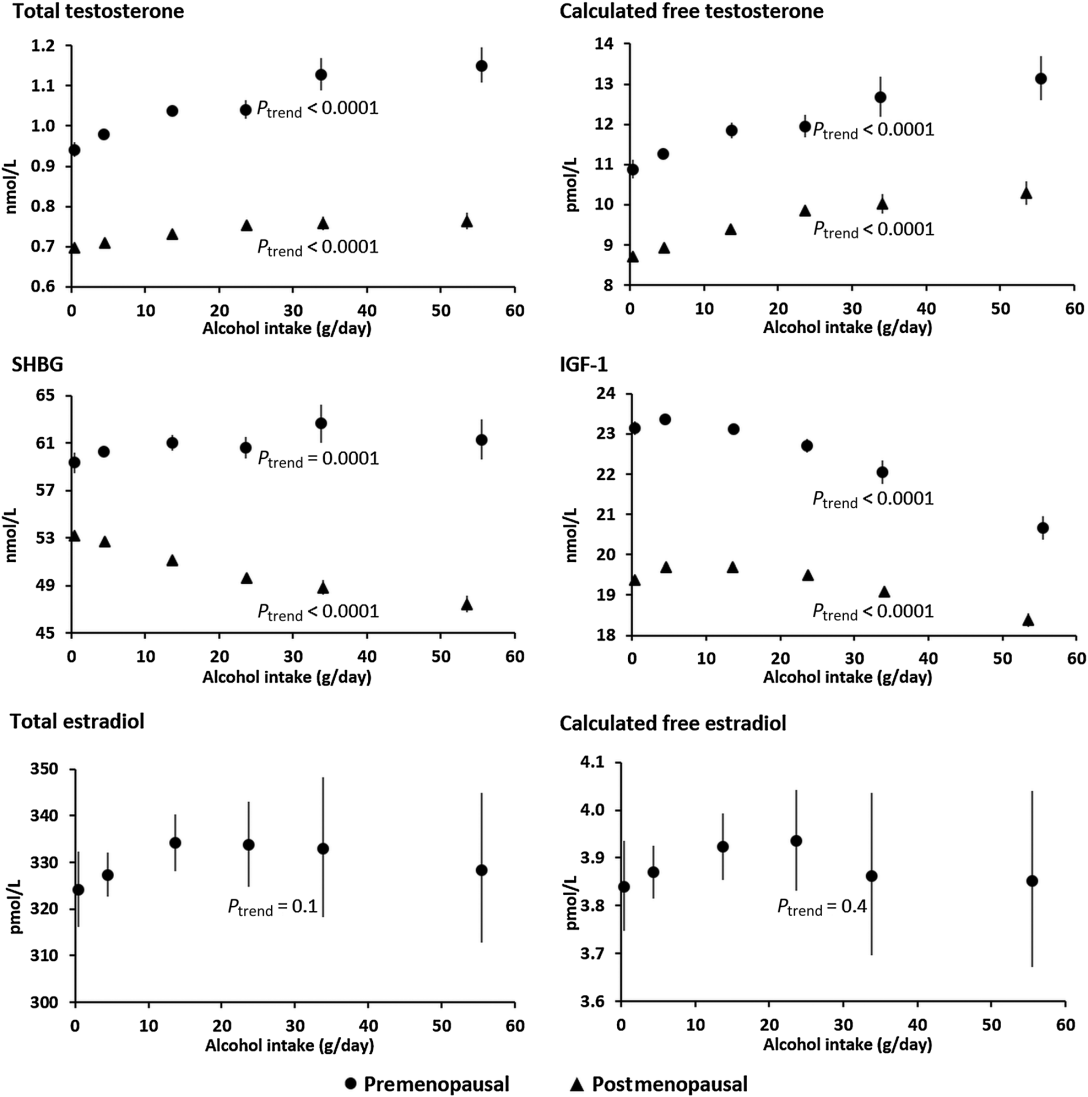
Geometric mean concentrations of hormones and SHBG by categories of habitual alcohol intake in pre- and postmenopausal women. Geometric mean concentrations adjusted for age, ethnicity, smoking, BMI, presence of endocrine disorder(s), age at menopause (postmenopause), time of day of sample collection and deprivation, and plotted against the mean alcohol intake within each category.

In both pre- and postmenopausal women, habitual alcohol intake was positively associated with testosterone concentrations (Table 2; Fig. 1; Supplementary Table S2). The geometric mean concentrations of total testosterone in the highest alcohol intake category (≥40 g/day) were 0.21 nmol/L (premenopausal) and 0.07 nmol/L (postmenopausal) higher than in those who consumed <1 g/day, and the concentrations of calculated free testosterone were 2.3 pmol/L (premenopausal) and 1.6 pmol/L (postmenopausal) higher (Fig. 1; Supplementary Table S2). The association of alcohol intake with total testosterone was significantly greater in premenopausal than in postmenopausal women (P_heterogeneity_ < 0.0001).

**Table 2.** Associations of habitual alcohol intake (per 10 g/day increment) with hormones and SHBG in pre- and postmenopausal women.

	*N*	Age-adjusted percent change (95% CI)^a^	Multivariable-adjusted percent change (95% CI)^b^	*P*_trend_	*P*_heterogeneity_
Total testosterone					
Premenopausal	28,180	4.2 (3.6–4.9)	3.9 (3.3–4.5)	<0.0001	<0.0001
Postmenopausal	120,309	1.7 (1.3–2.1)	2.3 (1.8–2.7)	<0.0001	
Calculated free testosterone					
Premenopausal	28,180	3.5 (2.8–4.2)	3.5 (2.8–4.2)	<0.0001	0.4
Postmenopausal	120,309	2.2 (1.7–2.6)	3.9 (3.5–4.4)	<0.0001	
SHBG					
Premenopausal	28,180	1.2 (0.7–1.7)	0.7 (0.2–1.1)	0.002	<0.0001
Postmenopausal	120,309	−0.8 (−1.0 to −0.5)	−2.4 (−2.6 to −2.2)	<0.0001	
IGF-1					
Premenopausal	28,180	−1.7 (−1.9 to −1.5)	−1.9 (−2.1 to −1.7)	<0.0001	<0.0001
Postmenopausal	120,309	−0.4 (−0.5 to −0.2)	−0.8 (−0.9 to −0.6)	<0.0001	
Total estradiol					
Premenopausal	28,180	0.3 (−0.5 to 1.0)	0.5 (−0.3 to 1.3)	0.2	—
Calculated free estradiol					
Premenopausal	28,180	−0.3 (−1.0 to 0.5)	0.2 (−0.5 to 1.0)	0.6	—

^a^Adjusted for age.

^b^As above + adjusted for ethnicity, smoking, BMI, presence of endocrine disorder(s), age at menopause (postmenopause), time of day of sample collection and deprivation. *P*_trend_ and *P*_heterogeneity_ were calculated from this model.

Similar associations were observed for intakes of alcohol from wine, beer or cider, and spirits or liqueur (Supplementary Table S3). In premenopausal women, there were no significant differences in the associations by smoking status (Supplementary Table S4), by age (Supplementary Table S5), or by phase of the menstrual cycle (Supplementary Table S6). In postmenopausal women, the association for calculated free testosterone was greater in current smokers (*P*_heterogeneity_ = 0.03; Supplementary Table S4).

### SHBG

In premenopausal women, there was a weak positive association between habitual alcohol intake and SHBG concentration (Table 2; Fig. 1; and Supplementary Table S2). Compared with women who consumed <1 g/day, those who consumed ≥40 g/day had a 2.0 nmol/L higher concentration of SHBG (Fig. 1; Supplementary Table S2). The concentration was 0.7% (95% CI: 0.2%–1.1%) higher per 10 g/day increment in alcohol intake (Table 2). In contrast, there was a significant inverse association with SHBG in postmenopausal women (Table 2; Fig. 1; Supplementary Table S2). Women who consumed ≥40 g/day had a 5.7 nmol/L lower concentration of SHBG than those who consumed <1 g/day (Fig. 1; Supplementary Table S2). The concentration was 2.4% (95% CI: 2.2%–2.6%) lower per 10 g/day increment in alcohol intake (Table 2). The associations observed in pre- and postmenopausal women were significantly different (*P*_heterogeneity_ < 0.0001).

Similar associations were observed for intakes of alcohol from wine, beer or cider, and spirits or liqueur (Supplementary Table S3). The positive association in premenopausal women was greater in never smokers (*P*_heterogeneity_ = 0.02), whereas the inverse association in postmenopausal women was greater in current smokers (*P*_heterogeneity_ = 0.005; Supplementary Table S4). There were no significant differences in the associations by age (Supplementary Table S5) or by phase of the menstrual cycle (Supplementary Table S6).

### IGF-1

In both pre- and postmenopausal women, there was a significant inverse association between habitual alcohol intake and IGF-1 concentration (Table 2; Fig. 1; Supplementary Table S2). The concentration was 2.5 nmol/L and 1.0 nmol/L lower, respectively, in pre- and postmenopausal women who consumed ≥40 g/day compared with those who consumed <1g/day (Fig. 1; Supplementary Table S2). Per 10 g/day increment in alcohol intake, the concentration was 1.9% (95% CI: 1.7%–2.1%) lower in premenopausal women and 0.8% (95% CI: 0.6%–0.9%) lower in postmenopausal women (Table 2). The association was significantly greater in premenopausal women (*P*_heterogeneity_ < 0.0001).

Similar associations were observed for intakes of alcohol from wine, beer or cider, and spirits or liqueur (Supplementary Table S3). In premenopausal women, there were no significant differences in the associations by smoking status (Supplementary Table S4), by age (Supplementary Table S5), or by phase of the menstrual cycle (Supplementary Table S6). The association in postmenopausal women was greater in those aged under 60 years (*P*_heterogeneity_ = 0.01; Supplementary Table S5).

### Total and calculated free estradiol

The estradiol results were available for premenopausal women only. There was no significant overall association with habitual alcohol intake (Table 2; Fig. 1; Supplementary Tables S2–S5). However, in subgroup analyses by phase of the menstrual cycle, a significant positive association was observed in the early and mid-luteal phases (*P*_heterogeneity_ = 0.046 for total estradiol and 0.06 for calculated free estradiol; Supplementary Table S6); per 10 g/day increment in alcohol intake, the concentration of total estradiol was 1.9% (95% CI: 0.2%–3.6%) and 2.4% (95% CI: 0.7%–4.2%) higher, respectively, in the early and mid-luteal phases, and that of calculated free estradiol was 1.8% (95% CI: 0.1%–3.5%) higher in both phases.

### Sensitivity analyses

Further adjustment for diet, parity and time since last OCP/HRT use did not have any material effect on the findings. Similar associations were observed after excluding women with self-reported endocrine disorders, premenopausal women with irregular menstrual cycles, and premenopausal women with a concentration of estradiol that was below the reportable range (Supplementary Table S7). The associations were slightly weaker after excluding women with a concentration of testosterone that was below the reportable range.

### Alcohol intake in the 24 hours before blood sampling

This analysis was restricted to a subsample of 4,751 pre- and 18,754 postmenopausal women who completed the Oxford WebQ questionnaire on the day of recruitment. Supplementary Table S8 presents characteristics of these women. The associations with 24-hour alcohol intake were slightly weaker compared with habitual alcohol intake (Supplementary Table S9). There were no significant differences in the associations by time of day of sample collection (Supplementary Table S10).

## Discussion

In this study, involving over 160,000 women, there were significant associations of habitual alcohol intake with serum concentrations of hormones and SHBG, with evidence of heterogeneity by menopausal status.

### Total and calculated free testosterone

We found a positive association between habitual alcohol intake and testosterone concentrations in both pre- and postmenopausal women, as observed in previous collaborative reanalyses of data from seven prospective studies in premenopausal women (7) and 13 prospective studies in postmenopausal women (13). The stronger associations in premenopausal women have also been reported in a previous analysis of the EPIC (European Prospective Investigation into Cancer and Nutrition) cohort (22).

An acute elevation in testosterone concentrations after alcohol intake has been reported in some intervention studies (23–25), although no significant change was observed in others (26, 27). However, in the current analysis, the associations with 24-hour alcohol intake were weaker than those for habitual intake; we may have had limited power to detect a short-term effect because alcohol is mostly consumed in the evening and the half-life of testosterone is short (20–100 minutes; ref. 28).

The physiologic mechanisms underlying the observed associations between alcohol intake and testosterone concentrations are not well understood. It has been proposed that alcohol may increase testosterone concentrations by stimulating its secretion from the ovaries and adrenal glands or by inhibiting its metabolism in the liver (29).

### SHBG

We found a weak positive association between habitual alcohol intake and SHBG concentration in premenopausal women but a stronger inverse association in postmenopausal women. A similar positive association in premenopausal women was observed in the Nurses' Health Study II (30), but the association was not significant in a collaborative reanalysis of data from seven prospective studies (7). The evidence in postmenopausal women was more consistent; alcohol intake has been inversely associated with SHBG in a previous collaborative reanalysis of data from 13 prospective studies (13).

In this analysis, we did not find a stronger association of SHBG with 24-hour alcohol intake. Similarly, no significant change in SHBG concentration after alcohol intake was observed in earlier intervention studies (26, 27).

SHBG is synthesized in the liver, and its plasma concentration is regulated by estrogens, androgens, insulin, and other hormones (31) as well as by pro- and anti-inflammatory cytokines (32). It is possible that alcohol influences SHBG concentration by affecting hormonal balance, altering cytokine levels, increasing hepatic synthesis/release, or decreasing blood clearance (33, 34), but the exact mechanism is unknown.

### IGF-1

We found an inverse association between habitual alcohol intake and IGF-1 concentration in both pre- and postmenopausal women. A similar association has been shown in a previous collaborative reanalysis of data from 17 prospective studies (9), and a lower IGF-1 concentration in heavy alcohol consumers has been reported (35–37). A decline in the IGF-1 concentration after daily intake of alcohol for 2 to 3 months has also been shown in previous cross-over trials (38, 39).

An acute decline in IGF-1 concentration within hours after alcohol intake has been reported in a previous intervention study (40). As the half-life of IGF-1 is 16 hours or more (41), we hypothesized that we might see a bigger association with alcohol intake in the previous day, but it was weaker than that of habitual intake.

IGF-1 is synthesized in the liver, and its circulating concentration is regulated by growth hormones and to a lesser extent by insulin and nutritional status (42). Higher alcohol intake may decrease IGF-1 through its inhibitory effect on growth hormone secretion (43), liver dysfunction (35), and/or malnutrition.

### Total and calculated free estradiol in premenopausal women

Alcohol intake has been positively associated with estradiol concentrations in a previous collaborative reanalysis of data from seven prospective studies (7). We found no significant overall association with total or calculated free estradiol, but in our subgroup analyses by phase of the menstrual cycle, there was a positive association in the early and mid-luteal phases. Variations by cycle phase have also been reported but the results varied; a positive association with luteal estradiol was reported in the Nurses' Health Study II (30), but in an earlier cross-over trial, after daily intake of 30 g of alcohol for three consecutive menstrual cycles, a significant increase in estradiol concentration (27.5%) was observed only at mid-cycle (26).

We did not find any significant association of estradiol with 24-hour alcohol intake, but a short-term increase in concentration after alcohol intake has been reported in earlier intervention studies (44, 45). We may have missed this potential association because the half-life of estradiol is short (∼3 hours; ref. 46).

Alcohol may influence estradiol concentration by altering its metabolism and clearance (44, 45, 47) or by affecting aromatization of androgens to estrogens (48). The overall null finding in our analysis may be due to certain limitations in the data for estradiol. First, the chemiluminescent immunoassays used in the UK Biobank were not as sensitive as more specialized assays for estradiol (49), the assay CV (coefficient of variation) at the concentrations we observed was relatively high (∼15%), and serum concentrations were too low to be measured by the assay in 20% of premenopausal women; we included these women in our analyses by setting the concentration at three quarters of the minimum reportable value, but this approach would be expected to reduce statistical power. Second, because the date of the menstrual period following blood collection was not recorded, it was not possible to use backward dating to define cycle phase, which is a more reliable categorization (50).

### Potential role of hormones in alcohol-induced breast carcinogenesis

Alcohol has been judged to be a carcinogen for many cancer sites including female breast cancer (3). The increase in risk is of moderate size, for example, previous analysis of the Million Women Study reported that the relative risk of breast cancer was 1.12 per 10 g/day increment in alcohol intake (12).

In this analysis, we found positive associations between alcohol intake and testosterone concentrations, but on the basis of the results from our previous analysis of the UK Biobank data (10), the difference in total testosterone concentrations between women who consumed ≥40 g/day of alcohol and those who consumed <1 g/day (0.21 nmol/L higher in premenopausal women and 0.07 nmol/L higher in postmenopausal women) would only predict an approximately 1.3% (premenopausal) and 2.3% (postmenopausal) higher risk of breast cancer. For SHBG, we observed a weak positive association in premenopausal women and an inverse association in postmenopausal women; the latter could possibly contribute to the increase in risk in postmenopausal women by increasing the proportion of free estradiol. For IGF-I, the association with alcohol intake was inverse; therefore, it is not likely to explain the higher cancer risk associated with alcohol intake. For estradiol, we found positive associations with alcohol intake in the early and mid-luteal phases. The associations were modest in this analysis possibly due to the limitations of data on this hormone but given a bigger effect observed in an earlier cross-over trial, estradiol is likely to make some contribution to the increase in cancer risk associated with alcohol intake in premenopausal women.

Overall, our findings suggest that the hormones we assessed are unlikely to explain all of the association between alcohol and breast cancer risk, but we did not have a comprehensive assessment of all potentially relevant hormones; therefore, no firm conclusion can be drawn. The published evidence suggests a slightly bigger effect of alcohol intake on ER (estrogen receptor) positive breast cancer than on ER-negative breast cancer (51, 52), which may support the potential role of hormones in alcohol-induced breast carcinogenesis. However, alcohol intake has also been associated with some increase in the risk of ER-negative breast cancer (except ER-negative progesterone receptor–negative breast cancer; refs. 51, 52), suggesting that other nonhormonal mechanisms may also be relevant.

### Strengths and limitations

The main strength of this study is the very large sample size with hormones measured for the full cohort. The limitations of the data on estradiol and phase of the menstrual cycle have been discussed above. A similar limitation applies to the testosterone assay, for which 16% of women had values below the lower limit of detection; this would be expected to limit the statistical power. Other limitations include the cross-sectional design, use of self-reported alcohol intake, use of blood samples collected at a single point in time, the lack of data on other hormones such as progesterone and androgens other than testosterone, and the study sample comprising predominantly white women aged over 40 years.

### Conclusions

The findings from this large study confirm significant but modest associations of alcohol with hormones and SHBG. The hormones we assessed are unlikely to explain all of the effects of alcohol on breast cancer risk, suggesting that other hormonal and nonhormonal mechanisms may also be important.

## Authors' Disclosures

S. Tin Tin reports grants from Health Research Council of New Zealand during the conduct of the study. T.J. Key and G.K. Reeves report grants from Cancer Research UK during the conduct of the study.
